# Beyond Traditional Risk Scores: Precision Medicine for Comprehensive Cardiovascular Risk Assessment in Women and Men

**DOI:** 10.3390/jcm15218365

**Published:** 2026-10-27

**Authors:** José M. Gámez, David Vivas, María Goya, Vicente Pallarés-Carratalá, Maria A. Sánchez-Calavera

**Affiliations:** 1 Department of Cardiology, Hospital Universitario Son Llatzer, 07198 Palma de Mallorca, Spain; 2 Departamento de Medicina, University of the Balearic Islands, 07120 Palma de Mallorca, Spain; 3 CIBEROBN (CIBER Physiopathology of Obesity and Nutrition CB12/03/30038), Carlos III Health Institute, 28029 Madrid, Spain; 4 Department of Cardiology, Hospital Clínico San Carlos, 28040 Madrid, Spain; 5 Departamento de Medicina, Universidad Complutense de Madrid, 28040 Madrid, Spain; 6 Obstetrics and Gynecology, Vall D’Hebron Hospital Universitari, 08035 Barcelona, Spain; 7 Faculty of Medicine, Universitat Autònoma de Barcelona, 08193 Cerdanyola Del Vallès, Spain; 8 Department of Medicine, Universitat Jaume I, 12071 Castellon, Spain; 9 Department of Internal Medicine, Psychiatry and Dermatology, Universidad de Zaragoza, 50009 Zaragoza, Spain

**Keywords:** cardiovascular risk, sex differences, risk modifiers, precision medicine, life-course prevention, preconception health, clinical checklists, risk reclassification

## Abstract

**Background and Objectives**: Conventional 10-year cardiovascular risk estimation algorithms are insufficient because they fail to integrate non-traditional clinical, biological, reproductive, and psychosocial modifiers that exhibit marked sex differences. The objective of this position framework is to address this gap by developing structured, sex-stratified clinical checklists for routine clinical practice. **Methods**: An expert narrative review and literature screening were conducted across PubMed/MEDLINE, Embase, Cochrane, and 14 international clinical practice guidelines (2020–2026 window) to identify robust sex-stratified risk modifiers. The multidisciplinary working group (comprising cardiology, gynecology, and primary care) conducted iterative internal review rounds to synthesize evidence and structure two comprehensive clinical checklists across six operational domains. **Results**: Epidemiological evidence mandates a life-course prevention model starting prior to conception. In women, obstetric history (preeclampsia, gestational diabetes, preterm birth), gynecological milestones (PMOS, endometriosis), early menopause, and oncological therapies markedly elevate vascular risk. In men, persistent erectile dysfunction, functional hypogonadism, androgen deprivation therapy, and premature androgenetic alopecia serve as early indicators of vascular and metabolic risk. Advanced biomarkers (Lp(a) ≥ 50 mg/dL, ApoB > 100 mg/dL, persistent hs-CRP ≥ 2 mg/L) and social determinants of health further refine risk stratification. **Conclusions**: Incorporating these structured checklists into daily consultations complements validated population risk scores (SCORE2, PREVENT, QRISK3), mitigates systematic underdiagnosis—especially in women—and provides an actionable framework for personalized cardiovascular prevention.

## 1. Introduction and Objectives

Cardiovascular disease (CVD) remains the leading cause of morbidity and mortality globally, accounting for over 18 million annual deaths [1,2]. Despite decades of therapeutic advances, substantial under-recognition and under-treatment of cardiovascular risk persist worldwide. A primary driver of this clinical gap is a historical male-centric bias in clinical trial enrollment and diagnostic criteria, which has led to systematic diagnostic delays, lower therapeutic intensity, and poorer outcomes in women [3].

European and international guidelines advocate for systematic 10-year risk estimation in primary prevention using validated population engines such as SCORE2, SCORE2-OP, SCORE2-Diabetes, QRISK3, PREVENT, and LIFE-CVD2 [4,5,6]. However, these standard multivariable risk engines rely almost exclusively on classic major risk factors: age, biological sex, systolic blood pressure, smoking status, and blood lipid concentrations. Consequently, they routinely fail to capture critical non-traditional clinical, biological, reproductive, and psychosocial modifiers that exhibit profound sex-stratified impacts [7].

In women, the presentation of ischemic heart disease is frequently heterogeneous and atypical compared to men. Entities such as ischemia with non-obstructive coronary arteries (INOCA), myocardial infarction with non-obstructive coronary arteries (MINOCA), coronary microvascular dysfunction, and Takotsubo syndrome exhibit a substantially higher prevalence in women, challenging traditional obstructive coronary disease paradigms [3,7,8,9]. Furthermore, historical enrollment in major cardiovascular randomized controlled trials has averaged ≤37% female participation, leaving major evidence gaps regarding sex-specific therapeutic efficacy [10]. A recent multidisciplinary Delphi consensus among primary care physicians, cardiologists, and gynecologists confirmed persistent diagnostic and therapeutic inequities toward female patients across routine clinical settings [11].

Conversely, in men, standard primary care consultations also omit vital gender-specific vascular indicators. Routine examinations rarely evaluate erectile function, serum testosterone levels, hypogonadal symptoms, anabolic–androgenic steroid exposure, or androgen deprivation therapy (ADT) [12,13,14]. Yet these parameters provide early, highly sensitive markers of systemic endothelial dysfunction, microvascular damage, and cardiotoxic exposure. Additionally, psychosocial stressors—including depression, chronic anxiety, social isolation, intimate partner violence, and socioeconomic deprivation—exert profound cardiovascular impacts across both sexes, with distinct exposure patterns and vulnerability windows [3,15].

### The New Paradigm: Life-Course Prevention

Historically, cardiovascular prevention has operated as a reactive, late-stage strategy aimed at mitigating overt vascular damage once classic risk factors manifest in middle age. Contemporary epidemiological and molecular evidence mandates a fundamental paradigm shift toward proactive, ‘cradle-to-grave’ life-course prevention [16,17]. Under this life-course framework, cardiovascular vulnerability begins prior to conception. Paternal obesity, metabolic dysfunction, and toxic exposures induce transmissible epigenetic modifications in sperm that alter offspring lipid metabolism and vascular structure from childhood. During pregnancy, the placenta serves as a real-time metabolic and vascular stress monitor; placental vascular lesions (e.g., acute atherosis of spiral arteries) directly mirror maternal cardiometabolic vulnerability and correlate with elevated maternal cardiovascular risk up to decades post-partum [15].

To bridge the critical gap between guideline recommendations and clinical implementation, we designed two comprehensive, sex-stratified clinical checklists. Operating as structured, non-competing adjuncts to established risk engines, these tools ensure systematic screening for non-traditional modifiers, enabling timely risk reclassification and personalized primary prevention [12].

To conceptualize this clinical challenge, cardiovascular risk assessment can be likened to an iceberg: standard risk engines evaluate only the visible tip, leaving major sex-specific modifiers submerged (Graphical Abstract).

## 2. Methodology

This work is structured as an Expert Narrative Review and Clinical Position Framework. Fourteen clinical practice guidelines and reference consensus documents from major international societies (including the European Society of Cardiology [ESC], American College of Cardiology/American Heart Association [ACC/AHA], European Atherosclerosis Society [EAS], and European Menopause and Andropause Society [EMAS]) were thoroughly evaluated [4,18,19,20,21,22].

A comprehensive literature search was conducted across PubMed/MEDLINE, Embase, and Cochrane databases covering the publication window from January 2020 to January 2026, while retaining foundational historical citations for essential clinical context. The Boolean search strings utilized included targeted combinations of medical subject headings (MeSHs) and text words: (‘cardiovascular risk’ OR ‘cardiovascular risk modifier’ OR ‘risk enhancer’) AND (‘sex differences’ OR ‘gender-specific’ OR ‘female-specific’ OR ‘male-specific’) AND (‘checklist’ OR ‘screening tool’ OR ‘risk reclassification’).

Study selection and eligibility criteria: inclusion criteria comprised prospective cohort studies, randomized controlled trials, systematic reviews, meta-analyses, and international practice guidelines evaluating non-traditional cardiovascular risk modifiers in adult populations. Pediatric populations (<10 years) were excluded, whereas late adolescents and young adults (15–19 years) presenting with early metabolic or reproductive transitions (e.g., polyendocrine metabolic ovarian syndrome [PMOS], early metabolic syndrome) were included to address early life-course vulnerability.

Consensus and checklist development process: the multidisciplinary working group—comprising 5 panel members representing cardiology, gynecology, and primary care—conducted iterative internal consensus rounds. Panelists systematically reviewed candidate variables retrieved from the literature, evaluating each item based on three explicit criteria: (1) strength of epidemiological association (hazard ratio or odds ratio), (2) biological plausibility, and (3) feasibility of rapid administration in routine primary care and outpatient consultations. Candidate items achieving consensus were synthesized into two sex-stratified clinical checklists (Supplementary Material).

Target population and applicability gate: both checklists are intended exclusively for primary prevention in adults without established atherosclerotic cardiovascular disease (ASCVD), familial hypercholesterolemia, severe chronic kidney disease (eGFR < 30 mL/min/1.73 m^2^), or diabetes mellitus with target organ damage. Patients with these high-risk conditions automatically require intensive preventive therapy, bypassing secondary risk reclassification.

## 3. Results

### 3.1. Checklist Architecture and Classic Risk Factors as the Essential Starting Point

To translate complex evidence into daily practice, the two clinical checklists were structured into six operational domains: (1) classic risk factors and population scores, (2) advanced biomarkers for reclassification, (3) sex-specific modifiers and reproductive/andrological milestones, (4) autoimmune and oncological comorbidities, (5) psychosocial and gender determinants, and (6) current symptom screening. The checklists employ a rapid binary format (‘Yes’, ‘No’, ‘Not Evaluated’) engineered for completion within an estimated 5 to 7 min during routine medical encounters.

Baseline evaluation incorporates standard multivariable parameters required by population risk engines (SCORE2, SCORE2-OP, SCORE2-Diabetes, QRISK3, PREVENT, LIFE-CVD2) [4,5,6,18,19]: age, biological sex, smoking status, systolic blood pressure, total cholesterol, HDL-C, LDL-C, non-HDL-C, eGFR, and urinary albumin-to-creatinine ratio (UACR). Glycemic screening explicitly differentiates prediabetes from type 2 diabetes (HbA1c %). Visceral adiposity is assessed via waist circumference using sex-specific pathological thresholds (≥88 cm in women, ≥102 cm in men). Physical activity, dietary quality, and history of recreational drug or anabolic–androgenic steroid use are systematically documented.

A comprehensive, unpooled synthesis of all non-traditional risk modifiers—including their meta-analytic effect sizes, evidence hierarchy tiers, and recommended primary care actions—is summarized in Table 1.

### 3.2. Advanced Biomarkers for Risk Reclassification

Advanced biomarkers identify subclinical atherogenesis and vascular inflammation in apparently low-to-moderate-risk individuals [4]. Lipoprotein(a) [Lp(a)] measurement at least once in a lifetime is mandated; values ≥ 50 mg/dL (or ≥105 nmol/L) constitute an independent, genetically determined risk enhancer [20]. Apolipoprotein B (ApoB) quantifies atherogenic particle burden more accurately than LDL-C in insulin-resistant and hypertriglyceridemic states; per the 2025 ESC/EAS Focused Update, ApoB > 100 mg/dL denotes elevated risk and >120 mg/dL indicates high risk [22,34,39,40]. Persistent low-grade vascular inflammation is captured by high-sensitivity C-reactive protein (hs-CRP ≥ 2 mg/L on two separate measurements in the absence of acute infection) [34]. In patients at borderline or intermediate calculated risk with therapeutic uncertainty, Coronary Artery Calcium (CAC) scoring serves as a Class IIb tie-breaker image test [4], noting that a CAC score of 0 in young women does not rule out soft, non-calcified atheromatous plaque or microvascular dysfunction. Additional metabolic markers include thyroid dysfunction [41], serum uric acid (sUA > 6.0 mg/dL in women, >7.0 mg/dL in men) [42], and metabolic cardioprotection with GLP1Ra/SGLT2i [43].

### 3.3. Female-Specific Risk Modifiers

Pregnancy represents a physiological vascular and metabolic stress test. Adverse pregnancy outcomes (APOs)—including preeclampsia and eclampsia (hazard ratio (HR) 1.8–2.2) [23,25], gestational hypertension, gestational diabetes (HR 1.8) [24], preterm birth (<37 weeks), recurrent miscarriages (≥2), fetal growth restriction, and placental abruption—unmask latent vascular vulnerability decades before clinical manifest disease. Gynecological conditions such as polycystic ovary syndrome (recently renamed polyendocrine metabolic ovarian syndrome [PMOS]) [26,44] and endometriosis (HR 1.2–1.4) [27] carry chronic metabolic and systemic inflammatory burdens. Additional critical female modifiers include history of infertility or assisted reproductive technology [28], cardiotoxic breast cancer therapies (anthracyclines, anti-HER2 agents, thoracic radiation) [32,45], autoimmune diseases (systemic lupus erythematosus, rheumatoid arthritis, antiphospholipid syndrome, psoriasis) [35], migraine with aura [25], early menopause (<45 years; HR 1.50) [29,30], surgical menopause (bilateral oophorectomy), nulliparity, lack of breastfeeding, and exogenous hormone exposure (combined oral contraceptives, menopausal hormone therapy) [46].

### 3.4. Male-Specific Risk Modifiers

In men, the checklist systematically screens for hidden endothelial and microvascular damage through andrological and oncological fields. Persistent erectile dysfunction (≥3 months) serves as an independent vascular predictor, preceding overt coronary artery disease by 2 to 5 years and offering an invaluable primary prevention window [31]. Functional hypogonadism—defined by low morning fasting total testosterone (<200–300 ng/dL [≈6.9–10.4 nmol/L]) or calculated free testosterone (<50–65 pg/mL [≈5.0–6.5 ng/dL])—correlates strongly with visceral obesity, insulin resistance, and endothelial dysfunction [12,13,14]. In prostate cancer management, androgen deprivation therapy (ADT) induces severe metabolic syndrome, dyslipidemia, and cardiotoxicity (HR 1.38–1.65) [32]. Male factor infertility, obstructive sleep apnea (HR 1.45) [38], chronic kidney disease progression, occupational shift work, and early-onset male androgenetic alopecia (<30 years) represent additional key male modifiers, with androgenetic alopecia serving as an early phenotypic marker of hyperinsulinemia and metabolic syndrome [33,47].

### 3.5. Psychosocial Factors, Mental Health and Gender Diversity

Psychosocial stressors and social determinants of health exert profound, independent effects on cardiometabolic morbidity [36,37]. It is essential to distinguish biological sex (chromosomal, hormonal, anatomical traits) from gender determinants (social constructs, caregiving burdens, gender roles, socioeconomic constraints). While gender constructs affect all individuals, economic deprivation, lower education levels, and chronic caregiving stress disproportionately burden women, accelerating vascular aging [48].

Severe mental illness, major depressive disorder, and chronic anxiety increase coronary event risk by 30% to 50% (HR 1.30–1.50) [15]. Brief validated primary care instruments (Goldberg Anxiety Scale ≥5, Depression Scale ≥ 2; or international alternatives PHQ-9 and GAD-7) are integrated into both checklists. Furthermore, both tools mandate sensitive evaluation of intimate partner violence (IPV) or trauma history using validated screening tools (HARK/HITS). Chronic post-traumatic stress induces sustained hypothalamic–pituitary–adrenal axis activation, resulting in autonomic dysregulation, systemic low-grade inflammation, and endothelial injury. IPV screening requires absolute privacy, strict confidentiality, and established institutional referral pathways.

Finally, a distinct clinical consideration involves individuals receiving exogenous Gender-Affirming Hormone Therapy (GAHT). While broader clinical and ethical debates regarding cross-sex hormonal interventions remain ongoing, from a cardiovascular perspective, long-term non-physiological hormone exposure introduces significant exogenous endocrine burdens—including heightened risks of venous thromboembolism, ischemic stroke, and metabolic dysregulation—necessitating strict, cautious vascular monitoring based on active pharmacological regimens and baseline risk profiles [45,46].

### 3.6. Current Clinical Assessment and Symptom Screening

Systematic symptom screening ensures that patients presenting with unrecognized angina or cardiovascular symptoms are not mismanaged under a primary prevention protocol. In patients reporting chest pain or exertional dyspnea, applying structured evaluation tools such as the ESC 2024 Symptom Score [18] allows rapid clinical triage. The presence of active symptoms immediately shifts the consultation focus from 10-year population risk scoring to urgent diagnostic workup for obstructive or non-obstructive CAD.

## 4. Implications for Clinical Practice and Implementation

Integrating sex-specific checklists standardizes medical history-taking and minimizes clinical variability across healthcare settings. Clinical application follows a structured three-phase workflow:

- Phase 1: systematic recording of mandatory variables to compute baseline 10-year population risk scores (SCORE2, SCORE2-OP, SCORE2-Diabetes, QRISK3, PREVENT, or LIFE-CVD2).
- Phase 2: comprehensive evaluation of sex-specific modifiers, reproductive/andrological milestones, and psychosocial determinants.
- Phase 3: identification of critical risk enhancers to guide upward risk reclassification or selective imaging (CAC score) in borderline or low-to-moderate-risk scenarios.

Reclassification logic and avoidance of double-counting: non-traditional risk modifiers function as qualitative risk enhancers. When ≥1 major risk enhancer is identified in a patient categorized as low-to-moderate risk, clinicians should consider reclassifying the patient into a higher risk tier to initiate or intensify preventive interventions (e.g., lipid-lowering therapy or blood pressure optimization). To prevent double-counting or over-treatment, risk enhancers are evaluated qualitatively without adding cumulative points to baseline multivariable risk equations [4,48,49].

### Challenges, Ethical Considerations, and Digitalization

Successful primary care implementation depends heavily on electronic health record (EHR) integration. Smart EHR systems utilizing conditional activation logic can automatically expand relevant domains—such as opening a post-partum surveillance tab when a history of preeclampsia is checked, or triggering an endothelial evaluation module when persistent erectile dysfunction is recorded. Ethical considerations are paramount: inquiring about intimate partner violence, mental health, or sexual function requires absolute privacy, clinician empathy, and clear institutional referral pathways [48,49].

## 5. Practical Proposal and Authors’ Recommendations

1. Dynamic risk scoring: use validated population engines (SCORE2, PREVENT, QRISK3) as the initial quantitative foundation, interpreting results dynamically alongside identified risk modifiers.
2. Comprehensive female screening: systematically document obstetric history (APOs), gynecological conditions (PMOS, endometriosis), menopausal age, and exogenous hormone exposure.
3. Mandatory male domain screening: systematically evaluate persistent erectile dysfunction (≥3 months), fasting morning testosterone (<200–300 ng/dL), ADT exposure, sleep apnea, and occupational shift work.
4. Biomarker reclassification: measure Lp(a) at least once in a lifetime (cut-off ≥50 mg/dL); evaluate ApoB (>100 mg/dL) and persistent hs-CRP (≥2 mg/L) in dyslipidemic or inflammatory states.
5. Selective imaging tie-breakers: utilize Coronary Artery Calcium (CAC) scoring in intermediate-risk patients when therapeutic decisions remain uncertain.
6. Psychosocial and mental health integration: screen for social determinants of health, depression/anxiety (PHQ-9/GAD-7), and intimate partner violence under strict confidentiality.

## 6. Future Research Directions and Limitations

### 6.1. Study Limitations and Implementation Feasibility

This work is an expert position proposal based on narrative review and consensus. Key limitations include: (a) the absence of prospective cohort validation regarding hard event reduction; (b) expert selection bias; (c) regional focus on European guidelines; and (d) potential risks of over-diagnosis. Feasibility evaluation indicates an item count of 25–30 fields with an estimated completion time of 5–7 min in primary care (optimizable via electronic health record conditional logic).

### 6.2. Staged Validation Roadmap

This position proposal provides a structured framework based on rigorous evidence synthesis and multidisciplinary expert consensus. Main limitations include the necessity for prospective validation in diverse primary care cohorts and the potential risk of over-diagnosis if reclassification rules are applied indiscriminately. A three-stage validation roadmap is outlined:

- Stage 1 (feasibility and usability): assess completion times, electronic EHR integration, missing data rates, and inter-observer reliability in primary care pilot centers.
- Stage 2 (process and patient-reported outcomes): measure appropriate preventive treatment initiation, patient-reported outcome measures (PROMs), quality of life improvement, and healthcare cost-effectiveness [48,49].
- Stage 3 (prognostic net benefit): evaluate incremental discrimination (C-statistic), Net Reclassification Improvement (NRI), and long-term hard event reduction over standard risk scores alone.

## 7. Conclusions

Cardiovascular risk assessment must evolve from static estimation focused exclusively on classic risk factors (the visible tip) toward a comprehensive, structured, sex-sensitive, and psychosocial-aware model.

Specific clinical checklists for women and men bring critical, routinely omitted modifiers to light, structuring history-taking and advancing equitable precision medicine from guidelines into daily practice, subject to future prospective validation.

## Figures and Tables

**Table 1:** Evidence hierarchy, effect sizes, and proposed actions for risk modifiers.

Risk Modifier Category	Specific Risk Modifier	Target Pop.	Evidence Tier *	Quantitative Effect Size (95% CI)	Primary Source	Proposed Clinical Action
Obstetric	Preeclampsia/Eclampsia	Women	Consolidated	HR 1.80–2.20 (1.60–2.50)	Parikh 2021 [23]	Post-partum metabolic follow-up; lifelong BP and renal surveillance
Obstetric	Gestational Diabetes	Women	Consolidated	HR 1.80 (1.40–2.20)	O’Kelly 2022 [24]	T2DM screening q1–3y; early lifestyle and weight intervention
Obstetric	APOs (Preterm, FGR, Loss)	Women	Consolidated	HR 1.30–1.50 (1.10–1.80)	Rabadia 2025 [25]	Recognize pregnancy as stress test; initiate primary prevention
Gynecological	PMOS/PCOS	Women	Consolidated	OR 1.50–1.80 (1.20–2.20)	Teede 2026 [26]	Screen insulin resistance, metabolic syndrome and dyslipidemia
Gynecological	Endometriosis	Women	Emerging	HR 1.20–1.40 (1.05–1.60)	Saad 2025 [27]	Longitudinal CVR monitoring; evaluate chronic inflammation
Gynecological	Female Infertility/ART	Women	Emerging	HR 1.15–1.35 (1.02–1.55)	Farland 2023 [28]	Incorporate reproductive history into early risk stratification
Hormonal	Premature/Early Menopause	Women	Consolidated	HR 1.50 (1.28–1.76)	Muka 2016 [29]	Intensify lipid and BP targets due to early estrogen loss
Hormonal	Surgical Menopause (Oophorectomy)	Women	Consolidated	HR 1.40 (1.18–1.65)	Maas 2021 [30]	Evaluate MHT indication; monitor lipid and metabolic shift
Vascular	Migraine with Aura	Women	Consolidated	HR 1.42 (1.21–1.66)	Rabadia 2025 [25]	Strict stroke and BP control; avoid combined estrogen OCs
Andrological	Persistent Erectile Dysfunction	Men	Consolidated	HR 1.44 (1.29–1.60)	Mostafaei 2021 [31]	2–5y window before CAD; evaluate vascular CVRFs and CAC
Andrological	Functional Hypogonadism	Men	Consolidated	HR 1.30–1.50 (1.10–1.80)	Tang 2024 [12]	Morning total (<200–300 ng/dL) & free testosterone re-check
Andrological	Male Infertility/Impaired Semen	Men	Emerging	HR 1.25 (1.05–1.50)	Tang 2024 [12]	Screen subclinical metabolic syndrome and vascular health
Oncological	Androgen Deprivation Therapy	Men	Consolidated	HR 1.38–1.65 (1.20–2.00)	Lyon 2022 [32]	Cardio-oncology surveillance for dyslipidemia and metabolic syndrome
Phenotypic	Early Male Alopecia (<30y)	Men	Emerging	OR 1.30–1.50 (1.00–1.90)	Girishma 2025 [33]	Phenotypic marker for opportunistic metabolic syndrome screening
Biomarker	Lipoprotein (a) ≥ 50 mg/dL	Both	Consolidated	HR 1.35 (1.20–1.52)	Matta 2025 [20]	Independent risk enhancer; measure once per lifetime
Biomarker	Apolipoprotein B > 100 mg/dL	Both	Consolidated	HR 1.38 (1.22–1.56)	Mach 2025 [22]	Guide lipid-lowering when LDL-C and TG are discordant
Biomarker	Persistent hs-CRP ≥ 2 mg/L	Both	Consolidated	HR 1.45 (1.25–1.68)	Di Fusco 2025 [34]	Reflects residual inflammatory risk; double-check infection
Autoimmune	Systemic Lupus Erythematosus	Both	Consolidated	HR 1.80 (1.45–2.23)	Durante 2015 [35]	Screen subclinical ASCVD; strict CVRF control
Autoimmune	Rheumatoid Arthritis	Both	Consolidated	HR 1.45 (1.28–1.64)	Durante 2015 [35]	Apply 1.5x multiplier to standard risk scores
Autoimmune	Psoriasis	Both	Consolidated	HR 1.28 (1.12–1.46)	Durante 2015 [35]	Monitor systemic inflammation and metabolic syndrome
Psychosocial	Intimate Partner Violence/Trauma	Both	Consolidated	HR 1.42 (1.21–1.66)	Ebong 2024 [36]	Confidential HARK/HITS screening; referral pathways
Psychosocial	Severe Mental Illness/Depression	Both	Consolidated	HR 1.35 (1.18–1.54)	Rajan 2020 [15]	Administer PHQ-9/GAD-7; integrate behavioral care
Psychosocial	Low Socioeconomic Status	Both	Consolidated	HR 1.30 (1.15–1.47)	Backholer 2017 [37]	Address social determinants of health and medication access
Comorbidity	Obstructive Sleep Apnea	Both	Consolidated	HR 1.45 (1.22–1.72)	Yeghiazarians [38]	Screen nocturnal hypoxia; CPAP therapy and BP monitoring

HR: hazard ratio; OR: odds ratio; * Evidence tier definitions: consolidated evidence indicates support from major clinical practice guidelines (e.g., ESC, ACC/AHA) and multiple independent prospective cohorts or meta-analyses establishing independent prognostic value. Emerging evidence indicates support from growing observational registries or biological plausibility, but without universal inclusion in baseline guidelines.

## Data Availability

No new data were created or analyzed in this study. Data sharing is not applicable to this article.

## Supplementary Materials

The following supporting information can be downloaded at: https://www.mdpi.com/article/10.3390/jcm15218365/s1, S1: Checklist for cardiovascular risk assessment in women, S2: Checklist for cardiovascular risk assessment in men.

## References

[1] Palaniappan L.P., Allen N.B., Almarzooq Z.I., Anderson C.A., Arora P., Avery C.L., Baker-Smith C.M., Bansal N., Currie M.E., Earlie R.S. (2026). 2026 Heart Disease and Stroke Statistics: A Report of US and Global Data from the American Heart Association. Circulation.

[2] Vervoort D., Wang R., Li G., Filbey L., Maduka O., Brewer L.C., Mamas M.A., Bahit M.C., Ahmed S.B., Van Spall H.G. (2024). Addressing the Global Burden of Cardiovascular Disease in Women: JACC State-of-the-Art Review. J. Am. Coll. Cardiol..

[3] Vogel B., Acevedo M., Appelman Y., Merz C.N.B., Chieffo A., A Figtree G., Guerrero M., Kunadian V., Lam C.S.P., Maas A.H.E.M. (2021). The Lancet women and cardiovascular disease Commission: Reducing the global burden by 2030. Lancet.

[4] Visseren F.L.J., Mach F., Smulders Y.M., Carballo D., Koskinas K.C., Bäck M., Benetos A., Biffi A., Boavida J.-M., Capodanno D. (2021). 2021 ESC Guidelines on cardiovascular disease prevention in clinical practice. Eur. Heart J..

[5] SCORE2 working group and ESC Cardiovascular Risk Collaboration (2021). SCORE2 risk prediction algorithms: New models to estimate 10-year risk of cardiovascular disease in Europe. Eur. Heart J..

[6] SCORE2-Diabetes Working Group, ESC Cardiovascular Risk Collaboration (2023). SCORE2-Diabetes: 10-year cardiovascular risk estimation in type 2 diabetes in Europe. Eur. Heart J..

[7] Ashcroft T., Bakker M., Anand A., Sattar N., A Maybin J., Kimenai D.M. (2025). Tailoring cardiovascular risk prediction to females. J. Endocrinol..

[8] Mehta L.S., Beckie T.M., DeVon H.A., Grines C.L., Krumholz H.M., Johnson M.N., Lindley K.J., Vaccarino V., Wang T.Y., Watson K.E. (2016). Acute Myocardial Infarction in Women: A Scientific Statement from the American Heart Association. Circulation.

[9] Wasniewski S., Kfouri Da Silva R., Capdeville S., Molina I.R., Virosta E., Cortés C.O., Díez-Villanueva P., Adeba A., Lera M.R., Muñoz L. (2025). Women’s health: An imAging-based cardiovascular risK-rEdUction Program (WAKE UP) study. Rationale and design. Front. Cardiovasc. Med..

[10] Gong I.Y., Tan N.S., Ali S.H., Lebovic G., Mamdani M., Goodman S.G., Ko D.T., Laupacis A., Yan A.T. (2019). Temporal Trends of Women Enrollment in Major Cardiovascular Randomized Clinical Trials. Can. J. Cardiol..

[11] Gámez J.M., Pedreira Pérez M., Fernández Olmo M.R., Laiz M.F., Inaraja V., Carratalá V.P. (2024). Multidisciplinary management of cardiovascular disease in women: Delphi consensus. Front. Cardiovasc. Med..

[12] Tang L., Chen M., Li J., Xu X., Pu X. (2024). Association of testosterone with myocardial infarction and severity of coronary artery disease among male patients. Int. J. Cardiol. Cardiovasc. Risk Prev..

[13] Alwani M., Yassin A., Talib R., Al-Qudimat A., Aboumarzouk O., Al-Zoubi R.M., Saad F., Haider K.S., Ansari A.A.J.D.P. (2021). Cardiovascular Disease, Hypogonadism and Erectile Dysfunction: Early Detection, Prevention and the Positive Effects of Long-Term Testosterone Treatment. Vasc. Health Risk Manag..

[14] Lincoff A.M., Bhasin S., Flevaris P., Mitchell L.M., Basaria S., Boden W.E., Cunningham G.R., Granger C.B., Khera M., Thompson I.M. (2023). Cardiovascular Safety of Testosterone-Replacement Therapy. N. Engl. J. Med..

[15] Rajan S., McKee M., Rangarajan S., Bangdiwala S., Rosengren A., Gupta R., Kutty V.R., Wielgosz A., Lear S., AlHabib K.F. (2020). Association of Symptoms of Depression with Cardiovascular Disease and Mortality in Low-, Middle-, and High-Income Countries. JAMA Psychiatry.

[16] Lloyd-Jones D.M., Allen N.B., Anderson C.A.M., Black T., Brewer L.C., Foraker R.E., Grandner M.A., Lavretsky H., Perak A.M., Sharma G. (2022). Life’s Essential 8: Updating and Enhancing the American Heart Association’s Construct of Cardiovascular Health. Circulation.

[17] Marx N., Federici M., Schütt K., Müller-Wieland D., A Ajjan R., Antunes M.J., Christodorescu R.M., Crawford C., Di Angelantonio E., Eliasson B. (2023). 2023 ESC Guidelines for the management of cardiovascular disease in patients with diabetes. Eur. Heart J..

[18] McEvoy J.W., McCarthy C.P., Bruno R.M., Brouwers S., Canavan M.D., Ceconi C., Christodorescu R.M., Daskalopoulou S.S., Ferro C.J., Gerdts E. (2024). 2024 ESC Guidelines for the management of elevated blood pressure and hypertension. Eur. Heart J..

[19] Ortiz A., Quiroga B., Díez J., Martín F.J.E.S., Ramirez L., Maraver M.P., Asensio M.L.M.-B., Arija J.Á.A., Fernández J.L.A.-O., Córdoba R. (2023). The Spanish Scientific Societies before the ESC 2021 guidelines on vascular disease prevention: Generalizing the measurement of albuminuria to identify vascular risk and prevent vascular disease. Nefrologia.

[20] Matta A., Taraszkiewicz D., Cougoul P., Lemozy S., Ferrières J. (2025). Association of high lipoprotein (a) level with carotid atherosclerosis and all-cause mortality. Am. J. Prev. Cardiol..

[21] Johannesen C.D.L., Langsted A., Nordestgaard B.G., Mortensen M.B. (2024). Excess Apolipoprotein B and Cardiovascular Risk in Women and Men. J. Am. Coll. Cardiol..

[22] Mach F., Koskinas K.C., Roeters van Lennep J.E., Tokgözoğlu L., Badimon L., Baigent C., Benn M., Binder C.J., Catapano A.L., De Backer G.G. (2025). 2025 Focused Update of the ESC/EAS Guidelines for the management of dyslipidaemias. Eur. Heart J..

[23] Parikh N.I., Gonzalez J.M., Anderson C.A.M., Judd S.E., Rexrode K.M., Hlatky M.A., Gunderson E.P., Stuart J.J., Vaidya D. (2021). Adverse Pregnancy Outcomes and Cardiovascular Disease Risk: Unique Opportunities for Cardiovascular Disease Prevention in Women: A Scientific Statement from the American Heart Association. Circulation.

[24] O’Kelly A.C., Michos E.D., Shufelt C.L., Vermunt J.V., Minissian M.B., Quesada O., Smith G.N., Rich-Edwards J.W., Garovic V.D., El Khoudary S.R. (2022). Pregnancy and Reproductive Risk Factors for Cardiovascular Disease in Women. Circ. Res..

[25] Rabadia S.V., Heimberger S., Cameron N.A., Shahandeh N. (2025). Pregnancy Complications and Long-Term Atherosclerotic Cardiovascular Disease Risk. Curr. Atheroscler. Rep..

[26] Teede H.J., Bahri Khomami M., Morman R., Laven J.S., Joham A.E., Costello M.F., Patil M., Rees D.A., Berry L., Cree M.G. (2026). Polyendocrine metabolic ovarian syndrome, the new name for polycystic ovary syndrome: A multistep global consensus process. Lancet.

[27] Saad M., Ansari I., Ibrahim Z.S., Batool R.M., Ahsan S.I., Arshad M.S., Collins P., Ahmed R. (2025). Increased risk of cardiovascular disease in women with endometriosis: A systematic review and meta-analysis. Eur. J. Obstet. Gynecol. Reprod. Biol..

[28] Farland L.V., Wang Y., Gaskins A.J., Rich-Edwards J.W., Wang S., Magnus M.C., Chavarro J.E., Rexrode K.M., Missmer S.A. (2023). Infertility and Risk of Cardiovascular Disease: A Prospective Cohort Study. J. Am. Heart Assoc..

[29] Muka T., Oliver-Williams C., Kunutsor S., Laven J.S.E., Fauser B.C.J.M., Chowdhury R., Kavousi M., Franco O.H. (2016). Association of Age at Onset of Menopause with Cardiovascular Outcomes: A Systematic Review and Meta-analysis. JAMA Cardiol..

[30] Maas A., Rosano G., Cifkova R., Chieffo A., van Dijken D., Hamoda H., Kunadian V., Laan E., Lambrinoudaki I., Maclaran K. (2021). Cardiovascular health after menopause transition, pregnancy disorders, and other gynaecologic conditions: Consensus document. Eur. Heart J..

[31] Mostafaei H., Mori K., Hajebrahimi S., Abufaraj M., Karakiewicz P.I., Shariat S.F. (2021). Association of erectile dysfunction and cardiovascular disease: An umbrella review of systematic reviews and meta-analyses. BJU Int..

[32] Lyon A.R., López-Fernández T., Couch L.S., Asteggiano R., Aznar M.C., Bergler-Klein J., Boriani G., Cardinale D., Cordoba R., Cosyns B. (2022). 2022 ESC Guidelines on cardio-oncology in practice. Eur. Heart J..

[33] Girishma J., Sanjana A.S., Nagesh R., Okade R., Krithika G.M. (2025). Prevalence of Metabolic Syndrome and Cardiovascular Risk Factors in Early Onset Male Androgenetic Alopecia. J. Med. Sci. Health.

[34] Di Fusco S.A., Volpe M., Nardi F., Matteucci A., Aquilani S., Marino G., Aiello A., Colivicchi F. (2025). Reducing LDL-Cholesterol to Very Low Levels: Sailing Between Established Benefits and Potential Risks. High. Blood Press. Cardiovasc. Prev..

[35] Durante A., Bronzato S. (2015). The increased cardiovascular risk in patients affected by autoimmune diseases: Review of the various manifestations. J. Clin. Med. Res..

[36] Ebong I.A., Quesada O., Fonkoue I.T., Mattina D., Sullivan S., de Oliveira G.M.M., Spikes T., Sharma J., Commodore Y., Ogunniyi M.O. (2024). The Role of Psychosocial Stress on Cardiovascular Disease in Women: JACC State-of-the-Art Review. J. Am. Coll. Cardiol..

[37] Backholer K., Peters S.A.E., Bots S.H., Peeters A., Huxley R.R., Woodward M. (2017). Sex differences in the relationship between socioeconomic status and cardiovascular disease: Systematic review. J. Epidemiol. Community Health.

[38] Yeghiazarians Y., Jaffe H., Luosterinen M., Redline S., Brown D.L., El-Sherif N., Mehra R., Bozkurt B., Ndumele C.E., Somers V.K. (2021). Obstructive Sleep Apnea and Cardiovascular Disease: A Scientific Statement from the American Heart Association. Circulation.

[39] Vivas D., Escobar C., Cordero A., Fernández-Olmo R., Oterino A., Blanco-Echevarría A., Botana M., Escribano D., Freijo M.d.M., Martínez-Losas P. (2024). Uso de nuevas terapias hipolipemiantes en la práctica clínica. Consenso SEC/SEA/SEEN/SEMFYC/SEMERGEN/SEMG/SEN/SEACV/S.E.N. REC CardioClinics.

[40] Grundy S.M., Stone N.J., Bailey A.L., Beam C., Birtcher K.K., Blumenthal R.S., Braun L.T., De Ferranti S., Faiella-Tommasino J., Forman D.E. (2019). 2018 AHA/ACC Guideline on the Management of Blood Cholesterol. J. Am. Coll. Cardiol..

[41] Soetedjo N.N.M., Agustini D., Permana H. (2024). The impact of thyroid disorder on cardiovascular disease: Unraveling the connection and implications for patient care. IJC Heart Vasc..

[42] Xiong J., Shao W., Yu P., Ma J., Liu M., Huang S., Liu X., Mei K. (2022). Hyperuricemia Is Associated with the Risk of Atrial Fibrillation Independent of Sex: A Dose-Response Meta-Analysis. Front. Cardiovasc. Med..

[43] Sgarra L., Matteucci A., Di Monaco A., Potenza M.A., Desantis V., Nacci C., Noviello C., Troisi F., Caccavo V.P., Mangini F. (2026). Impact of GLP1Ra and SGLT2i on atrial fibrillation in overweight, non-diabetic patients: A meta-analysis of randomized evidence. Sci. Rep..

[44] Teede H.J., Tay C.T., Laven J., Dokras A., Moran L.J., Piltonen T.T., Costello M.F., Boivin J., Redman L.M., Boyle J.A. (2023). Recommendations from the 2023 International Evidence-based Guideline for the Assessment and Management of Polycystic Ovary Syndrome. Fertil. Steril..

[45] Gulati M., Mulvagh S.L. (2018). The connection between the breast and heart in a woman: Breast cancer and cardiovascular disease. Clin. Cardiol..

[46] Johansson T., Karlsson T., Bliuc D., Schmitz D., Ek W.E., Skalkidou A., Center J.R., Johansson Å. (2024). Contemporary menopausal hormone therapy and risk of cardiovascular disease: Swedish nationwide register based emulated target trial. BMJ.

[47] Dharam Kumar K.C., Kishan Kumar Y.H., Neladimmanahally V. (2018). Association of Androgenetic Alopecia with Metabolic Syndrome. Indian. J. Endocrinol. Metab..

[48] Matteucci A., Mariani M.V., Sgarra L., Bonanni M., Pierucci N., La Fazia V.M., Fedele S., Nardi F., Colivicchi F. (2026). Beyond successful atrial fibrillation ablation: Unmet patient needs, quality-of-life gap and patients’ perspective. Expert Rev. Cardiovasc. Ther..

[49] Ademi Z., Rodda S.E., Vivoda K., Hennessy S., Fenton O., Ware J.S., Global Heart Hub Manifesto (2025). Highlights from the Manifesto on the Health Economics of Cardiovascular Disease Prevention. Pharmacoeconomics.

