# Supplementary material for: Beyond Traditional Risk Scores: Precision Medicine for Comprehensive Cardiovascular Risk Assessment in Women and Men
Source: J Clin Med. 2026 Oct 27;15(21):8365. doi: 10.3390/jcm15218365 (PMC13764131; doi:10.3390/jcm15218365)
Supplement: Supplementary file 1 [file jcm-15-08365-s001.zip › SUP_1.pdf]

CHECKLIST FOR CARDIOVASCULAR RISK ASSESSMENT IN WOMEN

TARGET POPULATION & APPLICABILITY GATE: Intended exclusively for primary prevention in adult women WITHOUT established Atherosclerotic Cardiovascular Disease (ASCVD), Familial Hypercholesterolemia, severe Chronic Kidney Disease (eGFR <30 mL/min/1.73m^2), or Diabetes with target organ damage. Patients with these high-risk conditions automatically require intensive preventive therapy without secondary risk reclassification.

PATIENT GENERAL INFORMATION & CLASSIC RISK FACTORS

Date of Birth: ______________ Age: _____ years Race/Ethnicity: Caucasian ☐ Afro-descendant ☐ Asian ☐ Hispanic ☐ Other ☐

- Systolic BP / Diastolic BP: ______ / ______ mmHg | Hypertension Diagnosis / Treatment: Yes ☐ No ☐
- Diabetes Mellitus (Type 1 or Type 2): Yes ☐ No ☐ | Age at Diagnosis: _____ yrs | HbA1c: _____ %
- Prediabetes / Impaired Fasting Glucose: Yes ☐ No ☐
- Lipid Profile: Total Chol: _____ mg/dL | LDL-C: _____ mg/dL | HDL-C: _____ mg/dL | Triglycerides: _____ mg/dL
- On lipid-lowering therapy: Yes ☐ No ☐
- Apolipoprotein B (ApoB): _____ mg/dL (☐ >100 mg/dL elevated risk | ☐ >120 mg/dL high/very high risk per 2025 ESC/EAS)
- Lipoprotein(a) [Lp(a)]: _____ mg/dL or _____ nmol/L (☐ ≥50 mg/dL or ≥105 nmol/L independent risk enhancer)
- Anthropometry: Weight: _____ kg | Height: _____ cm | BMI: _____ kg/m^2
- Pathological Waist Circumference (≥88 cm in women): Yes ☐ No ☐
- Smoking status: Current smoker ☐ Former smoker ☐ Non-smoker ☐
- Illicit drug / Anabolic-androgenic steroid use: Yes ☐ No ☐
- Sedentary lifestyle (<150 min/week moderate exercise): Yes ☐ No ☐
- Adherence to Mediterranean / Healthy diet: Yes ☐ No ☐
- Premature family history of CVD (men <55 yrs, women <65 yrs): Yes ☐ No ☐
- Renal Function: eGFR: _____ mL/min/1.73m^2 | UACR: _____ mg/g (☐ >30 mg/g microalbuminuria)
- High-sensitivity C-reactive protein (hs-CRP): _____ mg/L (☐ ≥2 mg/L persistent on 2 tests)
- Hypothyroidism / Thyroid Dysfunction: Yes ☐ No ☐ | Serum Uric Acid: _____ mg/dL

II. FEMALE-SPECIFIC RISK MODIFIERS

- Age at Menarche: _____ yrs | Menopausal Status: Pre/Perimenopausal ☐ Postmenopausal ☐
- Age at Menopause Onset: _____ yrs | Early Menopause (<45 yrs, HR 1.50): Yes ☐ No ☐
- Type of Menopause: Natural ☐ Surgical / Iatrogenic (Bilateral Oophorectomy) ☐
- Parity: Nulliparous ☐ Parous ☐ (Births: ____) | Breastfeeding Duration: Never ☐ <6 mos ☐ ≥6 mos ☐
- Polycystic Ovary Syndrome (PCOS / PMOS): Yes ☐ No ☐ Not evaluated ☐
- Endometriosis (HR 1.2–1.4): Yes ☐ No ☐ Not evaluated ☐
- Infertility history or Assisted Reproductive Technology (ART) use: Yes ☐ No ☐ Not evaluated ☐
- Adverse Pregnancy Outcomes (APOs) - Preeclampsia / Eclampsia (HR 1.8–2.2): Yes ☐ No ☐
- Gestational Diabetes (HR 1.80): Yes ☐ No ☐ | Gestational Hypertension: Yes ☐ No ☐
- Preterm birth (<37 weeks) / Fetal Growth Restriction / Recurrent Miscarriages (≥2): Yes ☐ No ☐
- Combined Oral Contraceptives / Menopausal Hormone Therapy (MHT): Yes ☐ No ☐
- Migraine with Aura (HR 1.42): Yes ☐ No ☐ Not evaluated ☐

III. ASSOCIATED MEDICAL CONDITIONS & COMORBIDITIES

- Systemic Lupus Erythematosus (HR 1.80): Yes ☐ No ☐ Not evaluated ☐
- Rheumatoid Arthritis (HR 1.45): Yes ☐ No ☐ Not evaluated ☐

• Psoriasis (HR 1.28) / Antiphospholipid Syndrome: Yes ☐ No ☐ Not evaluated ☐

• Active Cancer diagnosis / History of Cardiotoxic Chemotherapy or Radiotherapy: Yes ☐ No ☐

• Obstructive Sleep Apnea-Hypopnea Syndrome (OSA, HR 1.45): Yes ☐ No ☐ Not evaluated ☐

IV. PSYCHOSOCIAL, MENTAL HEALTH & GENDER DETERMINANTS

• Low socioeconomic status / Economic deprivation: Yes ☐ No ☐

• Chronic caregiving strain / High psychological stress: Yes ☐ No ☐

• Depression / Anxiety diagnosis: Yes ☐ No ☐ | Goldberg Anxiety (≥5): _____ | Goldberg Depression (≥2): _____

• Alternative validated scales: PHQ-9 score: _____ | GAD-7 score: _____

• Intimate Partner Violence (IPV) / Trauma history (HARK/HITS confidential tool): Yes ☐ No ☐ Declined ☐

• Transgender / Gender-diverse individual on Gender-Affirming Hormone Therapy (GAHT): Yes ☐ No ☐

V. CURRENT CLINICAL EVALUATION (ACTIVE SYMPTOM SCREENING)

Note: Presence of active cardiovascular symptoms triggers immediate diagnostic workup for CAD, not primary risk scoring.

• Active Chest Pain / Anginal equivalent: Yes ☐ No ☐ (If YES, apply ESC 2024 Symptom Score: _____ / 3)

• Exertional dyspnea or unexplained exercise intolerance: Yes ☐ No ☐

• Other cardiovascular symptoms: Palpitations ☐ Syncope ☐ Lower limb edema ☐ Unexplained fatigue ☐

VI. RISK CALCULATION, RECLASSIFICATION & ACTION PLAN

Baseline 10-Year Score (SCORE2/SCORE2-OP/SCORE DIABETES): _____ % | Category: Low-to-Moderate ☐ High ☐ Very High ☐

Consider Reclassifying Risk Upward if ≥1 Major Risk Enhancer is Present in Low-to-Moderate Risk:

☐ Lp(a) ≥ 50 mg/dL (or ≥ 105 nmol/L) or ApoB > 100 mg/dL

☐ Persistent hs-CRP ≥ 2 mg/L or UACR > 30 mg/g

☐ Adverse Pregnancy Outcomes (Preeclampsia, Gestational Diabetes, Preterm birth)

☐ Early Menopause (<45 years) / Surgical Menopause (Bilateral Oophorectomy)

☐ Polycystic Ovary Syndrome (PMOS) / Endometriosis / History of Infertility

☐ Migraine with Aura (HR 1.42)

☐ Severe Psychosocial Burden / Intimate Partner Violence / Major Depression

☐ Autoimmune Disease (Lupus, RA, Psoriasis) / Cardiotoxic Chemotherapy

Coronary Artery Calcium (CAC) Score (tie-breaker in intermediate risk): _____

Action Plan: Statin Initiation/Intensification: Yes ☐ No ☐ | BP Target Adjustment: Yes ☐ No ☐ | Multidisciplinary Referral: Yes ☐ No ☐
